# Coupling between mean blood pressure and EEG in preterm neonates is associated with reduced illness severity scores

**DOI:** 10.1371/journal.pone.0199587

**Published:** 2018-06-22

**Authors:** Oksana Semenova, Gordon Lightbody, John M. O’Toole, Geraldine Boylan, Eugene Dempsey, Andriy Temko

**Affiliations:** 1 Department of Electrical and Electronic Engineering, University College Cork, Cork, Ireland; 2 Irish Center for Fetal and Neonatal Translational Research, University College Cork, Cork, Ireland; 3 Department of Pediatrics and Child Health, University College Cork, Cork, Ireland; Universita degli Studi di Pisa, ITALY

## Abstract

Hypotension or low blood pressure (BP) is a common problem in preterm neonates and has been associated with adverse short and long-term neurological outcomes. Deciding when and whether to treat hypotension relies on an understanding of the relationship between BP and brain functioning. This study aims to investigate the interaction (coupling) between BP and continuous multichannel unedited EEG recordings in preterm infants less than 32 weeks of gestational age. The EEG was represented by spectral power in four frequency sub-bands: 0.3–3 Hz, 3–8 Hz, 8–15 Hz and 15–30 Hz. BP was represented as mean arterial pressure (MAP). The level of coupling between the two physiological systems was estimated using linear and nonlinear methods such as correlation, coherence and mutual information. Causality of interaction was measured using transfer entropy. The illness severity was represented by the clinical risk index for babies (CRIB II score) and contrasted to the computed level of interaction. It is shown here that correlation and coherence, which are linear measures of the coupling between EEG and MAP, do not correlate with CRIB values, whereas adjusted mutual information, a nonlinear measure, is associated with CRIB scores (*r* = -0.57, *p* = 0.003). Mutual information is independent of the absolute values of MAP and EEG powers and quantifies the level of coupling between the short-term dynamics in both signals. The analysis indicated that the dominant causality is from changes in EEG producing changes in MAP. Transfer entropy (EEG to MAP) is associated with the CRIB score (0.3–3 Hz: *r* = 0.428, *p* = 0.033, 3–8 Hz: *r* = 0.44, *p* = 0.028, 8–15 Hz: *r* = 0.416, *p* = 0.038) and indicates that a higher level of directed coupling from brain activity to blood pressure is associated with increased illness in preterm infants. This is the first study to present the nonlinear measure of interaction between brain activity and blood pressure and to demonstrate its relation to the initial illness severity in the preterm infant. The obtained results allow us to hypothesise that the normal wellbeing of a preterm neonate can be characterised by a nonlinear coupling between brain activity and MAP, whereas the presence of weak coupling with distinctive directionality of information flow is associated with an increased mortality rate in preterms.

## Introduction

Prematurity is the leading cause of death in children under the age of five [1] with more than 1 million children dying each year due to the complications of preterm birth. In addition, premature birth may also contribute to the development of different diseases in adulthood [2–4]. Hypotension, or low blood pressure (BP), is a common problem in preterm babies, particularly in the first 72 hours after delivery. It may cause decreased cerebral perfusion, resulting in impaired oxygen delivery to the brain [5]. The criteria which defines hypotension has not been clearly set [1] and the decision on when and whether it should be treated remains disputed resulting in considerable variability in practice [6], [7]. Treatment often involves administration of volume expanders and inotropes with dopamine as a first-line agent when the mean arterial pressure (MAP) (in mm Hg) falls below the gestational age (GA) in weeks [8]. This approach however is not supported by any robust scientific evidence [9]. At the same time, excessive intervention in order to treat hypotension in preterm infants has been associated with adverse outcomes, including brain injury [10]. Often, preterm infants with low BP have no biochemical or clinical signs of shock, and thus may not require any treatment. In this case a “permissive hypotension” approach, which implies careful observation without intervention may well be appropriate [11]. The ability to assess brain activity as a surrogate marker of adequate oxygen delivery may be an important adjunct to decision making in newborns with low BP. As such, preterm infants whose brain function is potentially impacted by low BP, may require treatment.

EEG and Near Infrared Spectroscopy (NIRS) are commonly used technologies to assess the ‘brain health’ of a newborn. EEG provides information about electrical cortical activity in the neonate [7]; NIRS allows continuous monitoring of cerebral oxygen saturation in the brain. Both methods are non-invasive and provide a real-time insight into brain function. Deciding when and whether to treat hypotension relies on our understanding of the relation between BP, oxygenation and brain activity. However, little is known about this relationship in preterm infants as these signals are rarely recorded simultaneously and the extraction and investigation of the complex measures of signal interaction and signal dynamics have not been explored.

Several studies have tried to establish the relationship between EEG activity and BP. Shah et al. [12] identified that BP and EEG energy were associated with flow in the superior vena cava in the first 12h of life. However, West et al. [13] found no association between superior vena cava flow and EEG energy. Increased oxygen extraction has been related to spontaneous activity transients observed in the EEG during the first 6h of life [14]. The levels of BP which result in abnormal cerebral activity, as quantified by EEG spectral features and peripheral blood flow measured with NIRS were studied in 35 very low birth weight infants in [5], where it was reported that a low BP (below 23 mm Hg) caused an increase in EEG discontinuity and a decreased relative power of the delta band (0.5–3.5 Hz). Changes in preterm EEG spectral power with maturation were also observed in a study by Niemarkt et al [15]. Most of these studies were performed on short EEG recordings utilizing only a single summary measure of the BP and EEG computed from the whole recording.

Detection of relationships and the quantification of interactions between physiological systems can be carried out in different ways, with classical linear methods, coherence and correlation being the most common [16]. Given two time series, the latter measures the level of linear coupling in the time domain, whereas the former quantifies the interaction between signals in the frequency domain. These measures capture linear relationships only and therefore fail to detect nonlinear coupling between the signals.

In contrast, our study hypothesises that a nonlinear measure of interaction between BP and EEG may be more sensitive to adverse health conditions than linear methods. In this work, the linear interaction between EEG and BP is quantified by classical coherence and correlation measures, while nonlinear coupling is computed based on mutual information (MI). Hypothesising that differences in coupling is indicative of preterm wellbeing, we test the association between this dynamic coupling and an illness risk score. There are a number of different neonatal scoring systems [17]; we evaluated the clinical risk index for babies (CRIB II) [18]. CRIB II is an improved version of CRIB and is used in neonatal intensive care for risk adjustment [11] including neonatal mortality risk prediction [18]. Compared to other risk scores, CRIB II (further referred to as CRIB) is found to have an improved discrimination power when assessing mortality risk for very low birth weight infants (<1500g) [19].

## Materials and methods

### Experimental design

**Fig 1:**
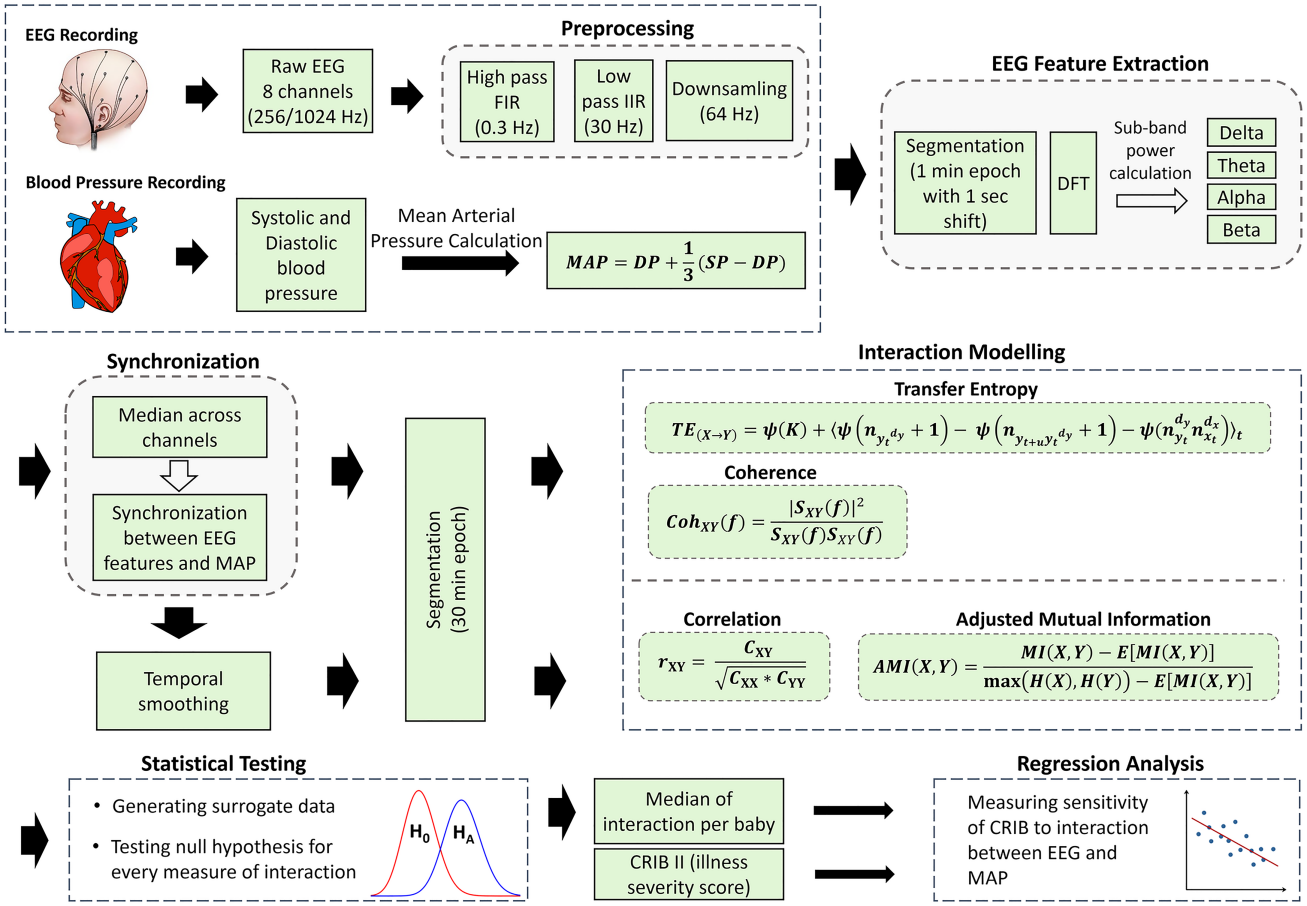
Overview of linear and nonlinear modelling of interaction between EEG and BP signals.

An overview of the signal preprocessing, feature extraction and the modelling of the interaction between brain activity and BP is shown in Fig 1. Measures of linear and nonlinear interaction between EEG and BP are computed. The computed values of coupling are summarized as the median across the whole recording for each newborn and then contrasted with the corresponding CRIB values. A regression line is fitted using the least squares method. Spearman’s rank correlation test (2-tailed) is used to conduct hypothesis tests on the correlation value. A correlation with *p < 0*.*05* is considered as statistically significant. Transfer entropy (TE) is also computed, to provide an insight into the directionality of the interaction between EEG and BP. The reliability of the obtained results is checked by testing an appropriate null hypothesis for every computed measure of interaction using surrogates. This is done in order to define whether a given empirical non-zero measurement of interaction is statistically different from zero.

### Dataset

**Fig 2:**
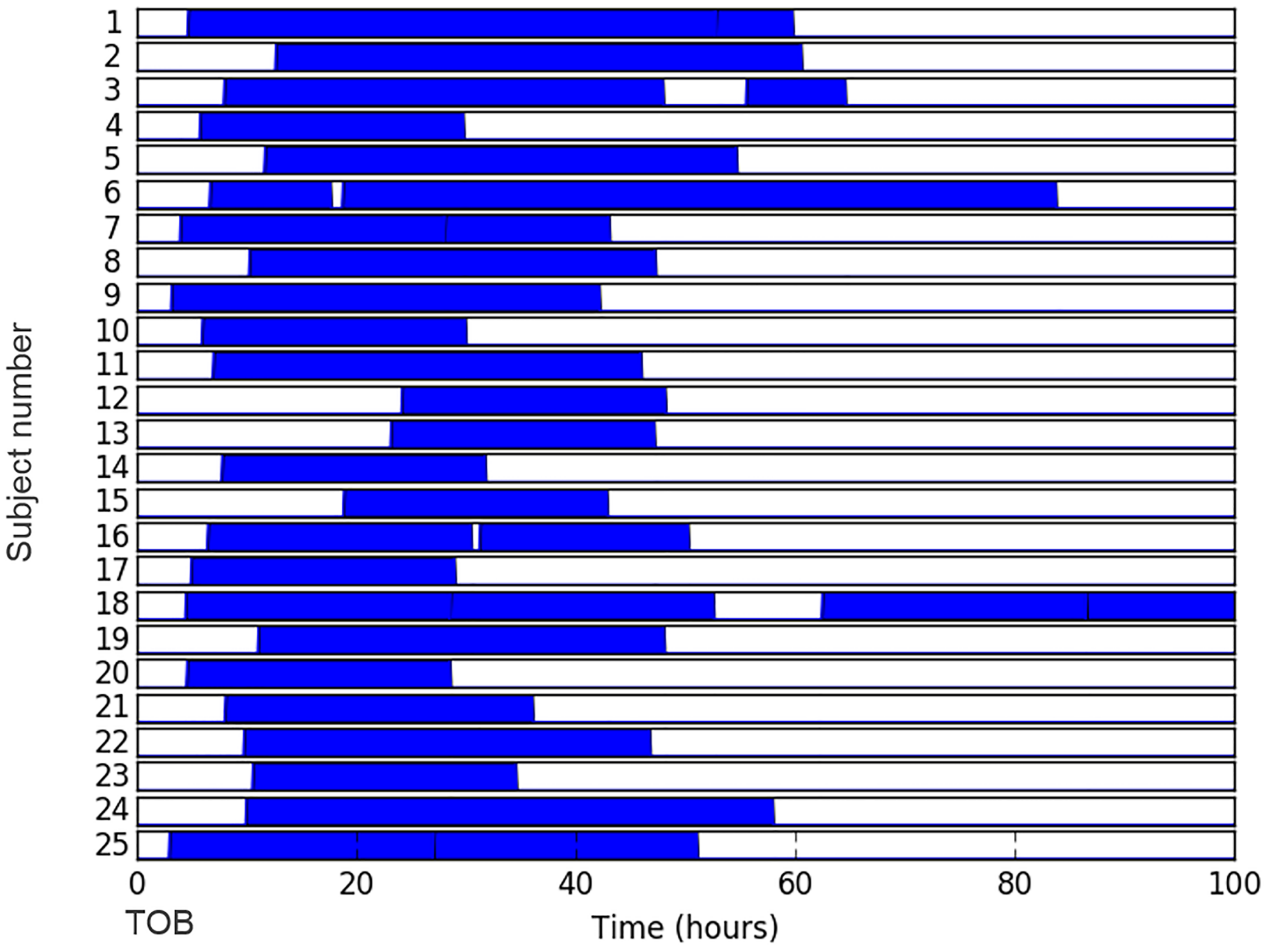
Schematic representation of duration and temporal location of recordings. Each recording is represented with respect to the time of birth (TOB) for each neonate.

The analysis is performed on a database of EEG data from 25 preterm infants < 32 weeks GA (range: 23–30 weeks) recorded at the neonatal intensive care unit of Cork University Maternity Hospital, Ireland. Clinical characteristics are provided in Table 1. The dataset included continuous multichannel EEG, simultaneous registration of BP and CRIB II scores. A Viasys NicOne video EEG machine (CareFusion Co., San Diego, USA) was used to record multi-channel EEG according to the well-established (in clinical practice) international 10–20 system of electrode placement, adjusted for neonates, with the analysis performed on the 8 bipolar channels: F4–C4, C4–O2, F3–C3, C3–O1, T4–C4, C4–Cz, Cz–C3 and C3–T3. The duration of recordings used in this study totals 957 hours (median = 37 hours, IQR = 24 to 48 hours). Fig 2 represents the duration and temporal location of each recording with the time of birth as a reference point. The EEG data were sampled at 256 Hz (21 subjects) and 1024 Hz (4 subjects).

**Table 1:** Clinical information, represented as median (IQR).

Subject #	GA (weeks)	BW (g)	CRIB	Apgar score 5 min	Gender	Umbilical cord pH	Recording duration (hours)
1	24	670	17	6	M	6.85	55
2	24	740	13	9	F	7.18	48
3	23	540	14	7	F	7.22	49
4	28	1040	7	9	F	7.3	24
5	25	730	12	7	F	7.29	43
6	30	1450	6	5	M	7.02	68
7	26	950	12	6	F	6.96	39
8	31	960	7	9	F	7.23	37
9	25	620	12	6	F	7.34	39
10	26	860	10	8	M	7.12	24
11	26	980	10	8	M	7.24	39
12	30	730	10	10	M	7.32	24
13	24	1240	5	9	F	7.15	24
14	28	1330	4	4	F	7.08	24
15	30	1000	7	10	F	7.24	24
16	28	650	9	7	F	7.22	43
17	28	980	8	8	F	7.16	24
18	28	1060	6	1	M	6.9	83
19	29	1230	7	9	M	7.27	37
20	24	620	13	9	F	7.27	24
21	30	800	10	8	M	7.28	28
22	28	980	8	10	F	7.26	37
23	30	1530	3	8	F	7.1	24
24	23	580	14	6	F	7.24	48
25	28	680	8	8	M	7.32	48
**Median** **(IQR)**	28 (25 to 29)	950 (680 to 1040)	9 (7 to 12)	8 (6 to 9)	64% (F)	7.23 (7.12 to 7.27)	37 (24 to 48)

**Fig 3:**
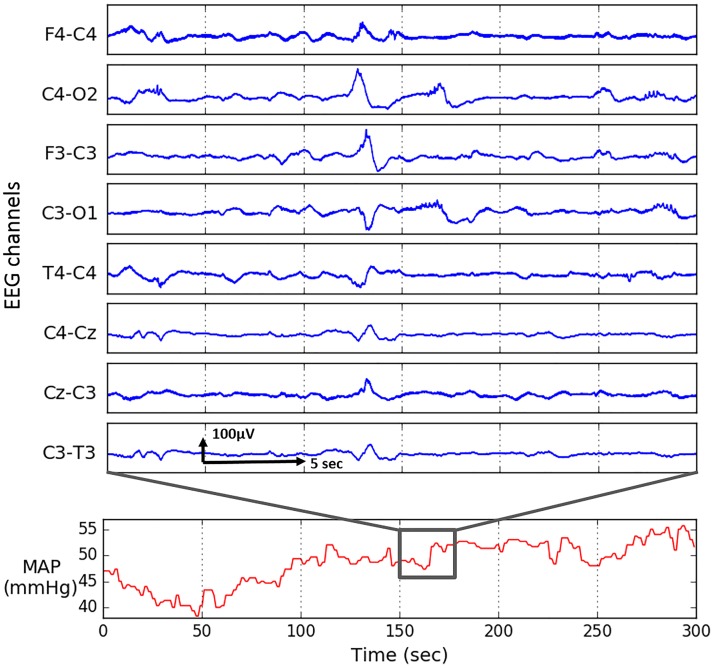
Five minutes of mean arterial pressure (MAP) and 30 seconds of eight-channel raw EEG recording.

Continuous invasive systolic (SP) and diastolic (DP) pressure monitoring was simultaneously performed via an umbilical arterial catheter using the Philips Intellevue MP70 machine which provides processed BP output at a rate of 1 per second. All infants were nursed supine. Positioning of the tip of the umbilical catheter in the descending aorta was confirmed by chest radiograph. BP is known to be a slowly evolving signal (Fig 3) and all important frequency components are within the 0–0.5 Hz band. Low frequency components of MAP were previously investigated for premature infants (0.005 Hz to 0.16 Hz) in [20] and adult population [21]. An example of a data segment is shown in Fig 3.

In this study the initial status of these preterm neonates is represented by the CRIB II score. This score is defined on a scale between 1 and 27 and depends on: sex, birth weight, GA, base excess and temperature at admission. Higher values are indicative of a greater risk of mortality corresponding to lower GA, birth weight and temperature at admission. This study had full ethical approval from the Clinical Research Ethics Committee of the Cork Teaching Hospitals. Parental written, informed consent was obtained for all newborns recruited for EEG monitoring studies. All data were anonymised.

### Preprocessing and feature extraction: EEG and BP

In order to measure the coupling between physiological signals it is necessary to derive informative values (features) that characterise the measured data. Prior to EEG feature extraction, the EEG signal is filtered to the range of 0.3–30 Hz and down-sampled to 64 Hz. The EEG was segmented into 1-minute epochs with 1-second shift. Each epoch of EEG was transformed into the frequency domain using the Discrete Fourier Transform (DFT). The power spectral density $X_{c}^{l}(f)$ for the *l*^th^ epoch of the *c*^th^ channel was subdivided into four frequency bands: 0.3–3 Hz, 3–8 Hz, 8–15 Hz and 15–30 Hz. This division slightly differs from the standard delta (0.5–3.5 Hz), theta (4–7.5 Hz), alpha (8–12.5 Hz) and beta (13–30 Hz) frequency bands. This was proposed as it better captures the brain dynamics [22], [23], [24] and accounts for rapid maturation changes [25] in the premature brain. The power of each sub-band was then calculated:

$$X_{c}^{l}(b)=\int_{f_{1}(b)}^{f_{2}(b)}X_{c}^{l}(f)df$$ (1)

where *f*_1_(*b*) − *f*_2_(*b*), for *b* = 1, 2, 3, 4 is one of the four frequency bands for *l*^*th*^ epoch of channel *c*. For every feature, the median value across all eight channels is calculated in order to reduce the effect of focal artefacts.

The DP and SP recorded every second were used to calculate the mean arterial pressure (MAP) as indicated in Fig 1. In clinical practice MAP is defined as the perfusion pressure of organs in the body and is commonly used to determine the intervention criteria for preterm neonates, including hypotension management [9]. At the same time both SP and DP are known to be less robust to errors than MAP [26]. The MAP signal was synchronized with features computed from the EEG by applying a moving average filter using the same epoch length and shift which was used for the EEG signal namely 1 minute epochs with 1 second shift.

While other studies mainly analysed preselected short EEG epoch, the main strength of the current work lies in the analysis performed on the long duration unedited multichannel EEG recordings (total of 957hours). The artifacts were removed automatically. In particular, the influence of EEG and MAP artefacts in our study was minimised using the following procedures. The usual amplitude-based thresholding of EEG was performed to automatically remove zero-signal and high amplitude artefact (e.g. eye blinking, electrode moved/disconnected). Also, the bipolar montage is used which is known to reduce the effect of many artefacts, e.g. ECG artefacts [27]. Spectral power features were extracted, which are known to be robust to low-amplitude artefacts. Each feature is summarised as a median across eight bipolar channels, thus making it robust to focal artefacts. Similarly, in order to get rid of artefacts in the BP signal, BP values less than 10 mmHg which occur when the pressure transducer is briefly disconnected or moved, were removed. Sharp and non-physiological changes in MAP were automatically eliminated by removing outliers in every 1-hour epoch. To remove the artifacts which are caused by intervention (e.g. due to infusion given through the line) 10 minutes of MAP before and after each intervention are ignored. Synchronisation assures that the segments which are ignored in EEG are also removed in BP and vice versa.

### Interaction modelling between EEG and BP features: Correlation and coherence

In this study, both linear and nonlinear measures of interaction of the previously synchronized EEG and MAP features were calculated over a 30-minute moving window with a 30 seconds shift. This window length allows one to focus on the short-term dynamics of both the EEG and MAP signals. Correlation is a way to determine the extent to which two variables co-vary linearly and it is defined as:

$$r_{xy}=\frac{C_{xy}\;}{\sqrt{C_{xx}C_{yy}}},$$ (2)

where ***C***_***xy***_ is the covariance between signals ***x*** and ***y***; ***C***_***xx***_ and ***C***_***yy***_ are the variance of signals ***x*** and ***y*** respectively. This measure captures information on the time coupling and waveform similarity between two signals. Correlation is sensitive to polarity and its values range from -1 to 1.

Unlike correlation which measures interaction in the time domain, the coherence measure has an advantage of showing the similarity of two variables for a chosen frequency. At each frequency ***f*** the coherence function ***Coh***_***xy***_ is defined by:

$${Coh}_{xy}(f)=\frac{|{S_{xy}(f)|}^{2}}{S_{xx}(f)S_{yy}(f)},$$ (3)

where ***S***_***xy***_**(*f*)** is the cross spectral density between ***x*** and ***y*; *S***_***xx***_**(*f*)** and ***S***_***yy***_**(*f*)** are the auto-spectral density of ***x*** and ***y***, respectively. Calculation of cross spectral density allows coherence to account for the possible lag between signals, whereas correlation is very sensitive to phase lag. The calculation of coherence also involves squaring the signal, thus producing values in range between 0 and 1.

Several window lengths were tried for the Welch periodogram computation. A segment width of 400 seconds was chosen as it resulted in the presence of distinctive peaks in the coherence plot. Every 30-minute window is then represented as a sum of coherence values within the window.

**Fig 4:**
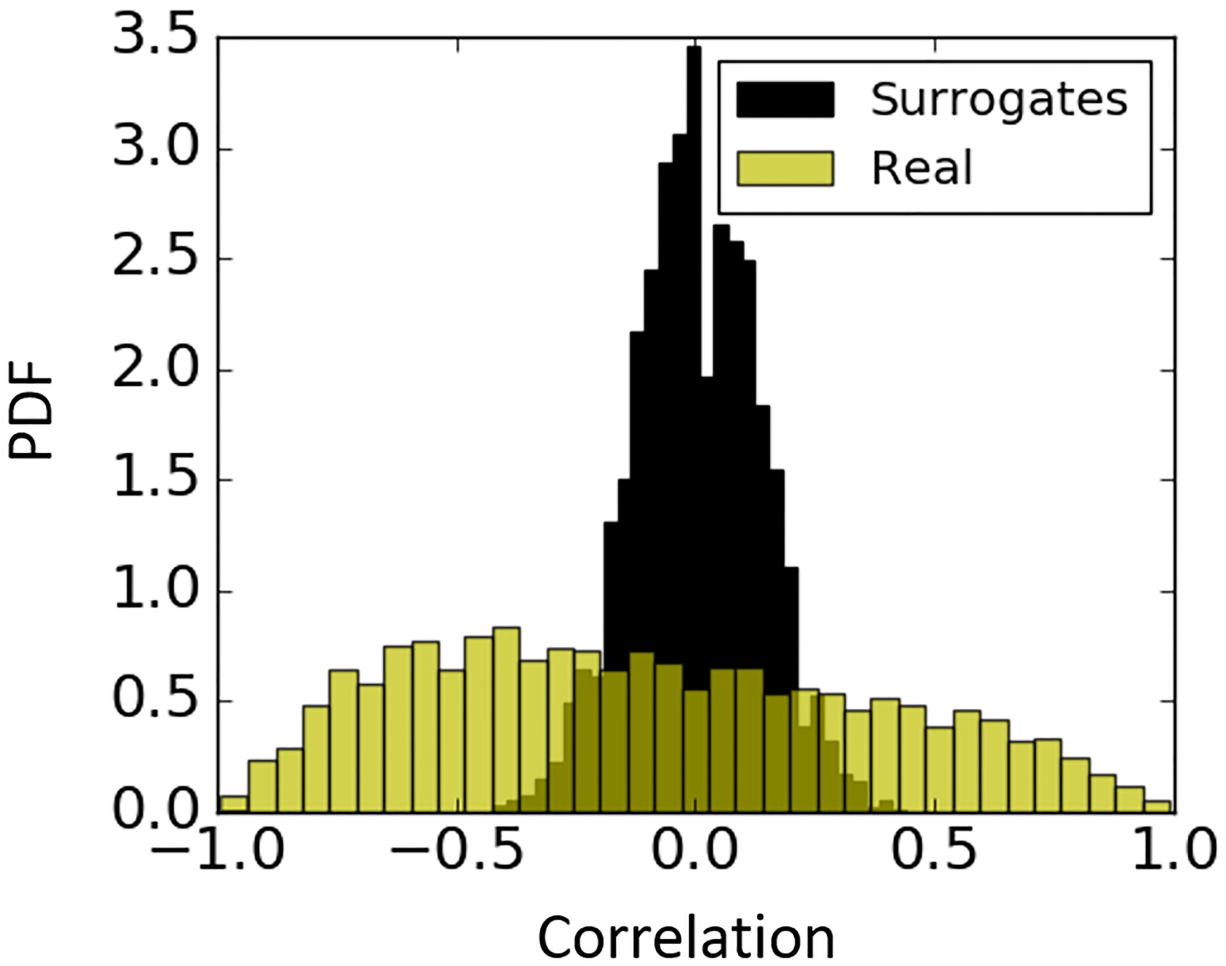
Probability density function (PDF) of Pearson correlation coefficient for surrogates and real data. Correlation for real data is quantified between MAP and EEG sub-energy (0.3–3 Hz) feature (yellow); correlation between corresponding randomly permuted surrogates of MAP and sub-band energy (0.3–3 Hz) feature (black).

In theory both the correlation and coherence between two unrelated sequences is equal to zero. However, in practice where interaction is empirically measured from a finite number of samples, a non-zero measurement is likely to result even if there is no relationship between signals. In order to check whether a given empirical non-zero measurements of correlation and coherence are statistically different from zero, a null hypothesis of no relationship between signals is tested. The significance of interaction between the original signals is then estimated against the distribution of interaction values obtained from shuffled surrogates. Surrogate data have been generated by the random permutation of the original values of MAP and sub-band powers. In other words, after shuffling both the ***X*** and ***Y*** sequences, instead of *p*(***x*** | ***y***), surrogate data is distributed as *p*(***x***). In the present study 100 shuffled surrogates were generated for every 30-minute epoch, while leaving the order of epochs unchanged. Fig 4 represents an example of the probability density function (PDF) of the null hypothesis for correlation. We can see that insignificant values range from -0.4 to 0.4 and therefore, should be ignored.

### Interaction modelling between EEG and BP features: Adjusted mutual information

Due to the likely complex relation between brain function and MAP, nonlinear methods of interaction were included [28]. MI is an information theoretic measure of dependency between two random variables defined as:

MI(X,Y)=H(X)+H(Y)−H(X,Y), (4)

where ***H***(***X***), ***H***(***Y***) are the Shannon entropies for sequences ***X*** and ***Y***. After substitution of:

$$H(X)=-\sum_{x}p(x){log}_{2}p(x)\;and\;H(Y)=-\sum_{y}p(y){log}_{2}p(y),$$ (5)

the MI is obtained as follows:

$$MI(X,Y)=\sum_{x\in X}\sum_{y\in Y}p(x,y){log}_{2}\left(\frac{p(x,y)}{p(x)p(y)}\right),$$ (6)

where *p*(*x*), *p*(*y*) are the probabilities of an occurrence of a particular label, *x*, *y*, in the sequences ***X*, *Y***; *p*(*x*,*y*) = *p*(*x*)*p*(*y*|*x*), where *p(y|x)* is the probability that a label, *y*, occurs in sequence *Y*, given another label, *x*, occurred in sequence *X*. It is easy to show that if the sequences ***X*** and ***Y*** are independent, then *p(y|x) = p*(*y*), and the ratio term *p(x*,*y)/(p(x)p(y))* becomes 1 and the ***MI***(***X***, ***Y***) becomes 0.

**Fig 5:**
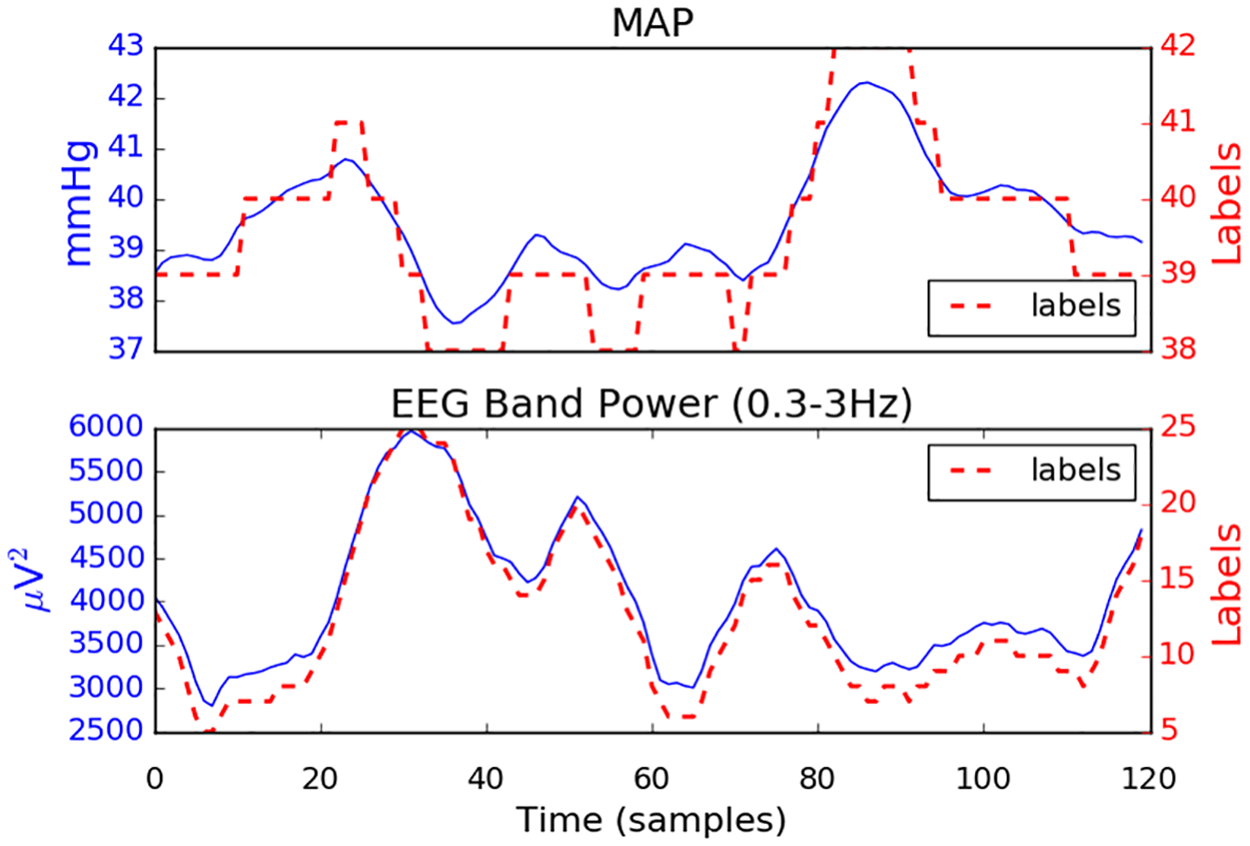
One hour of MAP and delta-band energy and its corresponding labels (dashed line). The shift of a 30 min window is 30 sec, which results in 120 values per hour.

In a similar manner to other information measures, the most common way for calculating MI from empirical data is to use histogram binning (labelling) in order to estimate the probability density distribution. The choice of the number of bins into which the two sequences *(****X*, *Y****)* are subdivided is important and may significantly affect the results. If there are too few, then it might be impossible to distinguish any structure in the distribution. Too many bins might result in occupation numbers of 0 which provides no meaningful information. Fig 5 illustrates an example of results of data labelling. The MAP trace is quantized into 5 labels whereas the trace of EEG delta-band powers is converted to 21 different labels.

**Fig 6:**
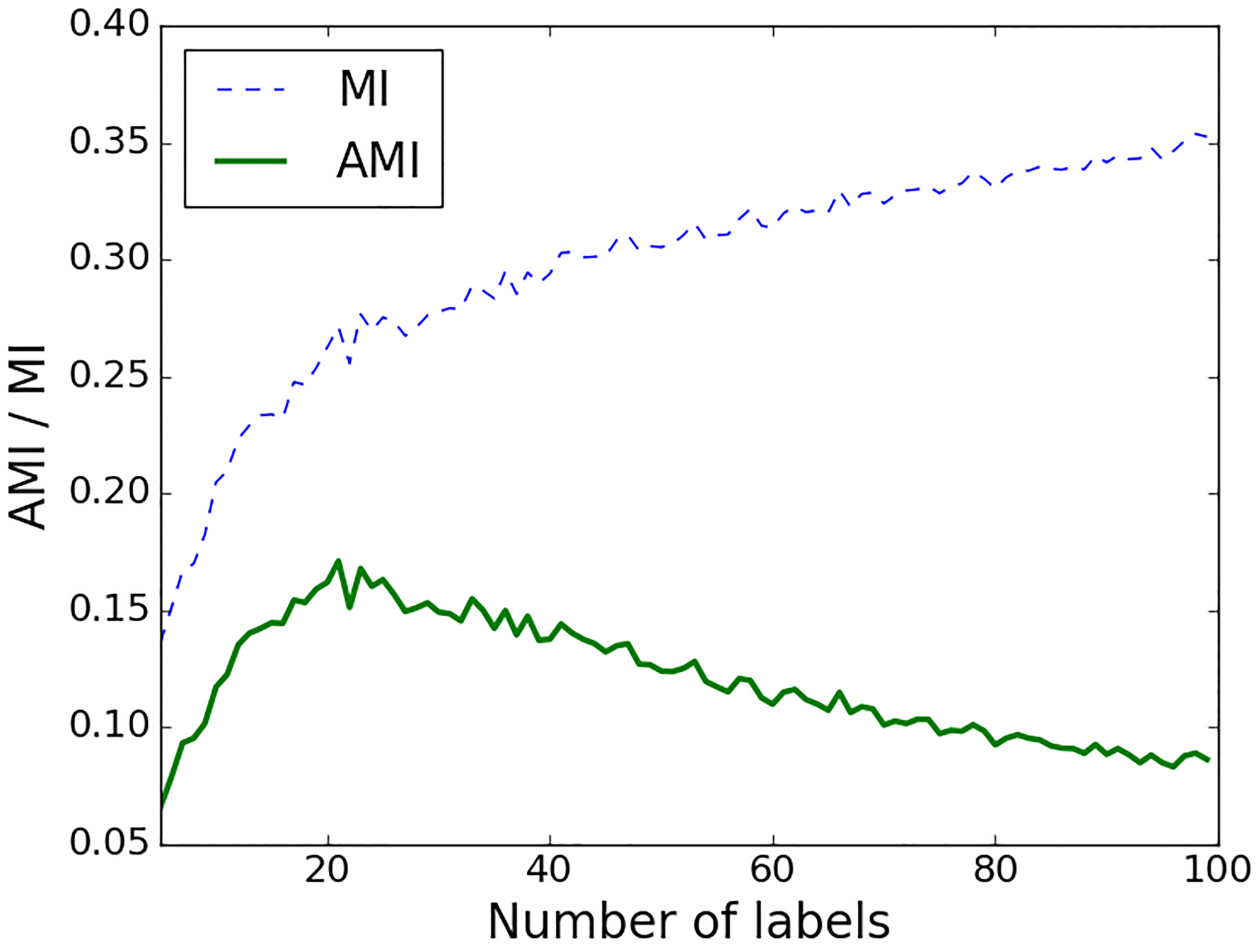
The effect of the number of labels on AMI and MI values. Interaction is calculated between MAP and the EEG power in the 0.3–3 Hz sub-band for one preterm infant. Every value is obtained as a mean across all epochs for a given number of labels.

In order to minimise the effect of the choice of the number of labels, the MI was calculated as an adjusted mutual information (AMI), which unlike conventional MI corrects the effect of agreement between two sequences which happens solely due to chance [29]. In particular, AMI accounts for the fact that MI tends to increase as the number of different labels increases, regardless of the actual amount of interaction between two sequences. This behaviour can be observed on Fig 6, where higher values of MI and lower AMI correspond to a higher number of labels into which the sequences are binned. After reaching some optimal number of labels, the values of AMI start to decrease, penalising the high MI caused by a higher number of labels only. Therefore, binning into an unreasonably high number of labels will result in very low values of AMI implying the absence of shared information between the two sequences beyond that of chance alone. The main advantage of AMI is that for the chosen number of bins, AMI measures the interaction that is adjusted for random chance; the conventional MI measure increases with the increase of random interactions which are caused by the high number of bins.

The AMI for two sequences (***X*, *Y***) is computed as:

$$AMI(X,Y)=\frac{MI(X,Y)-E[MI(X,Y)]}{max\left(H(X),H(Y)\right)-E[MI(X,Y)]},$$ (7)

with the expected value of the MI is defined as:

$$E[MI(X,Y)]=\sum_{x\in X}\sum_{y\in Y}\sum_{n_{xy}=max(1,a_{x}+b_{y}-N)}^{min\left(a_{x},b_{y}\right)}\frac{n_{xy}}{N}log(\frac{N\times n_{xy}}{a_{x}b_{y}})\times \;\frac{a_{x}!b_{y}!(N-a_{x})!(N-b_{y})!}{N!n_{xy}!(a_{x}-n_{xy})!(b_{y}-n_{xy})!(N-a_{x}-b_{y}+n_{xy})!}.$$ (8)

Here *a*_*x*_ and *b*_*y*_ are partial sums of the contingency table; *a*_*x*_ = ∑_*x*∈*X*_ *n*_*xy*_, *b*_*y*_ = ∑_*y*∈*Y*_ *n*_*xy*_, with *n*_*xy*_ as the number of labels common in ***X*** and ***Y***. AMI equals 1 when ***X*** and ***Y*** are identical (i.e. perfectly matched). For independent ***X*** and ***Y*** sequences the values of AMI is close to 0 (small negative values can also occur).

In this study, the MAP signal was binned into a number of labels that is equal to the range of integer MAP values. This number of labels prevents the artificial creation of dynamics in the MAP signal when the MAP fluctuates insignificantly (within 1 mmHg). The sub-band EEG energy was binned into 35 bins. This number has been chosen as the one that maximizes the values of AMI across all neonates.

**Fig 7:**
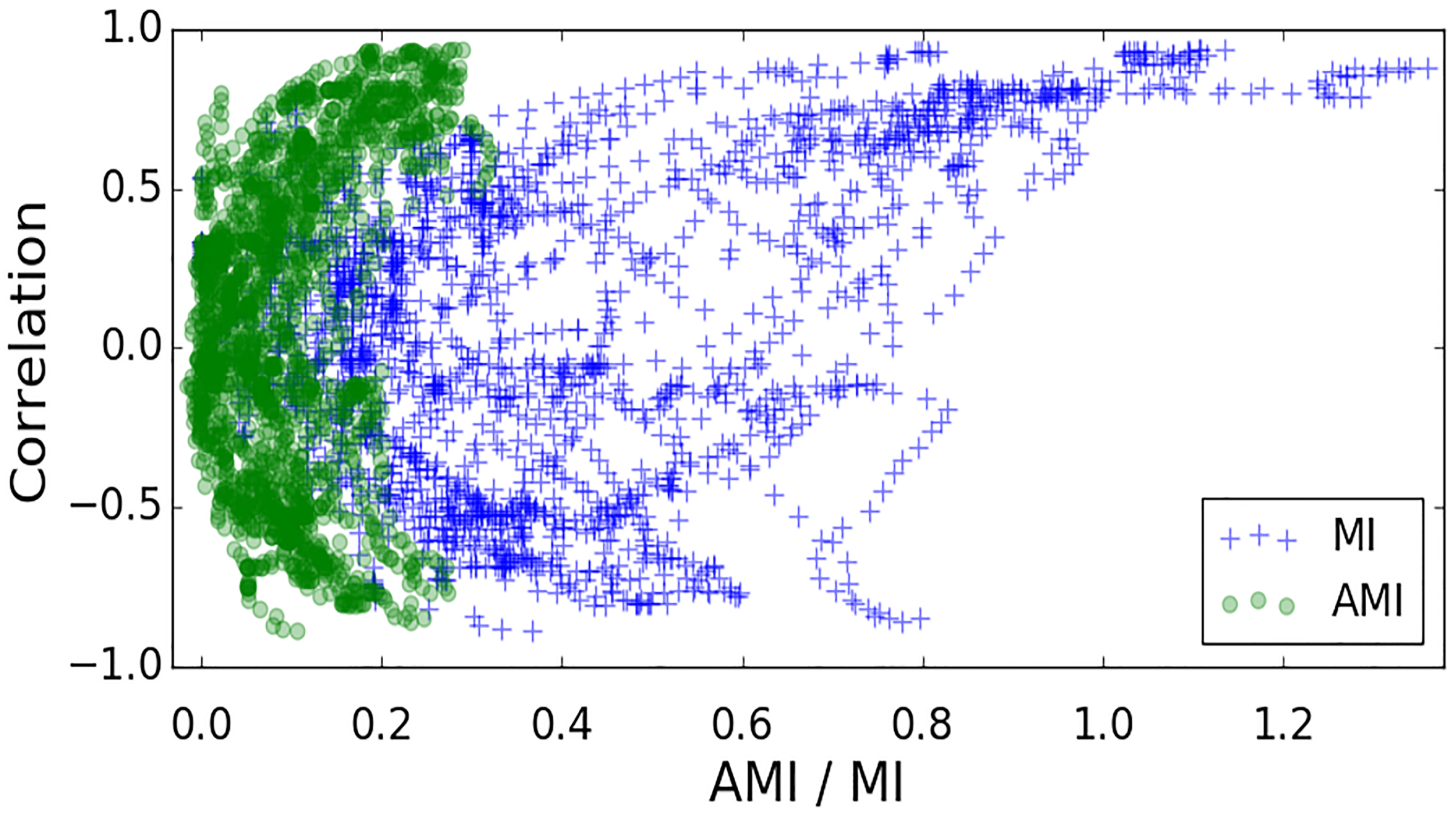
A scatter plot of conventional mutual information (MI), adjusted mutual information (AMI) and Pearson correlation. This plot represents measures of interaction between MAP and EEG (0.3–3 Hz) sub-band energy computed for each 30 min window from one preterm.

MI is a symmetric measure (*MI(X*, *Y) = MI(Y*, *X)*) and unlike correlation or coherence it quantifies both linear and nonlinear dependences. An example is shown in Fig 7, where the correlation, AMI and conventional MI are computed between MAP and EEG feature for one newborn. It can be seen that higher levels of both positive and negative correlation result in higher values of AMI and MI, where AMI values are shown to be more conservative as opposed to conventional MI.

#### Understanding AMI through simulation studies and surrogates

**Fig 8:**
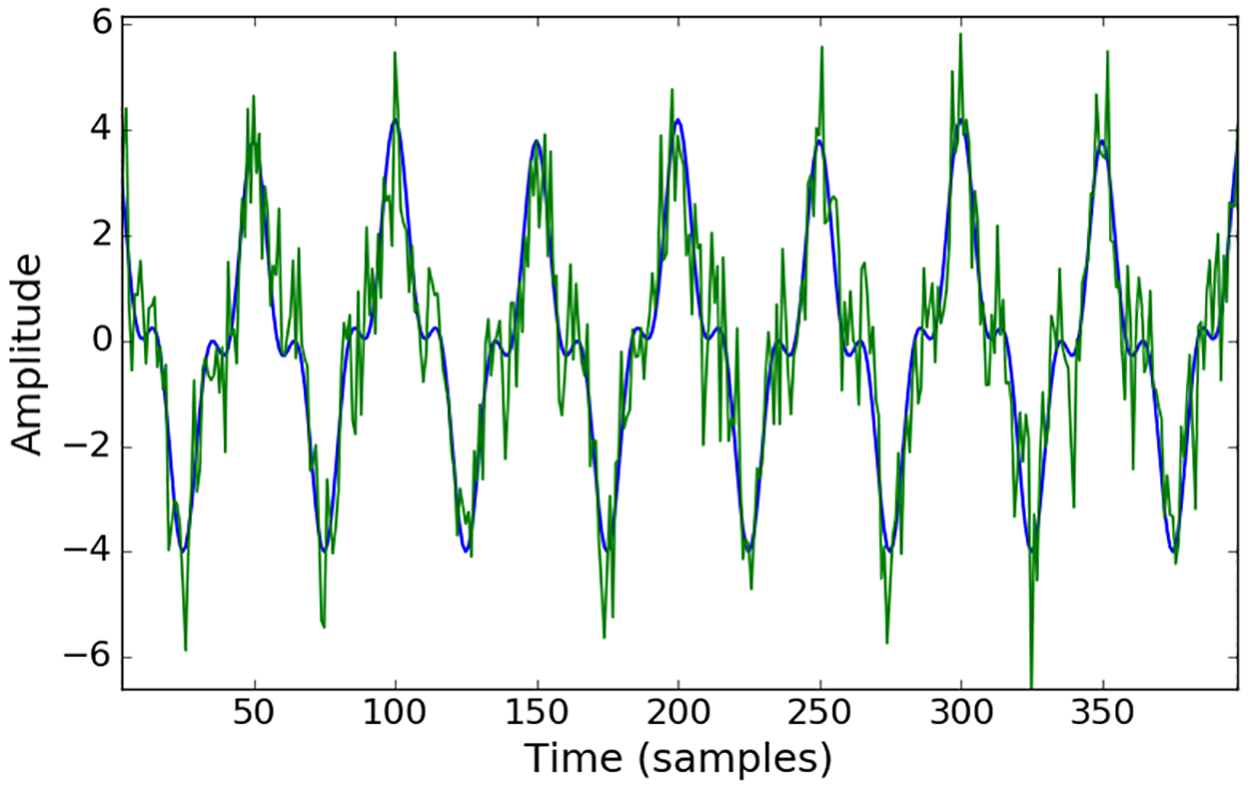
Trace of artificially generated toy data. *y*_0_ = 2.8 * *sin*(2*π* * 1000) + 1.2 * *sin*(2*π* * 3000*x*) + 0.2 * *sin*(2*π* * 500*x*) in blue and *y* = *y*_0_ + *N*(0,1) in green. Pearson correlation coefficient between ***y***_***0***_ and ***y*** is equal to 0.91.

**Fig 9:**
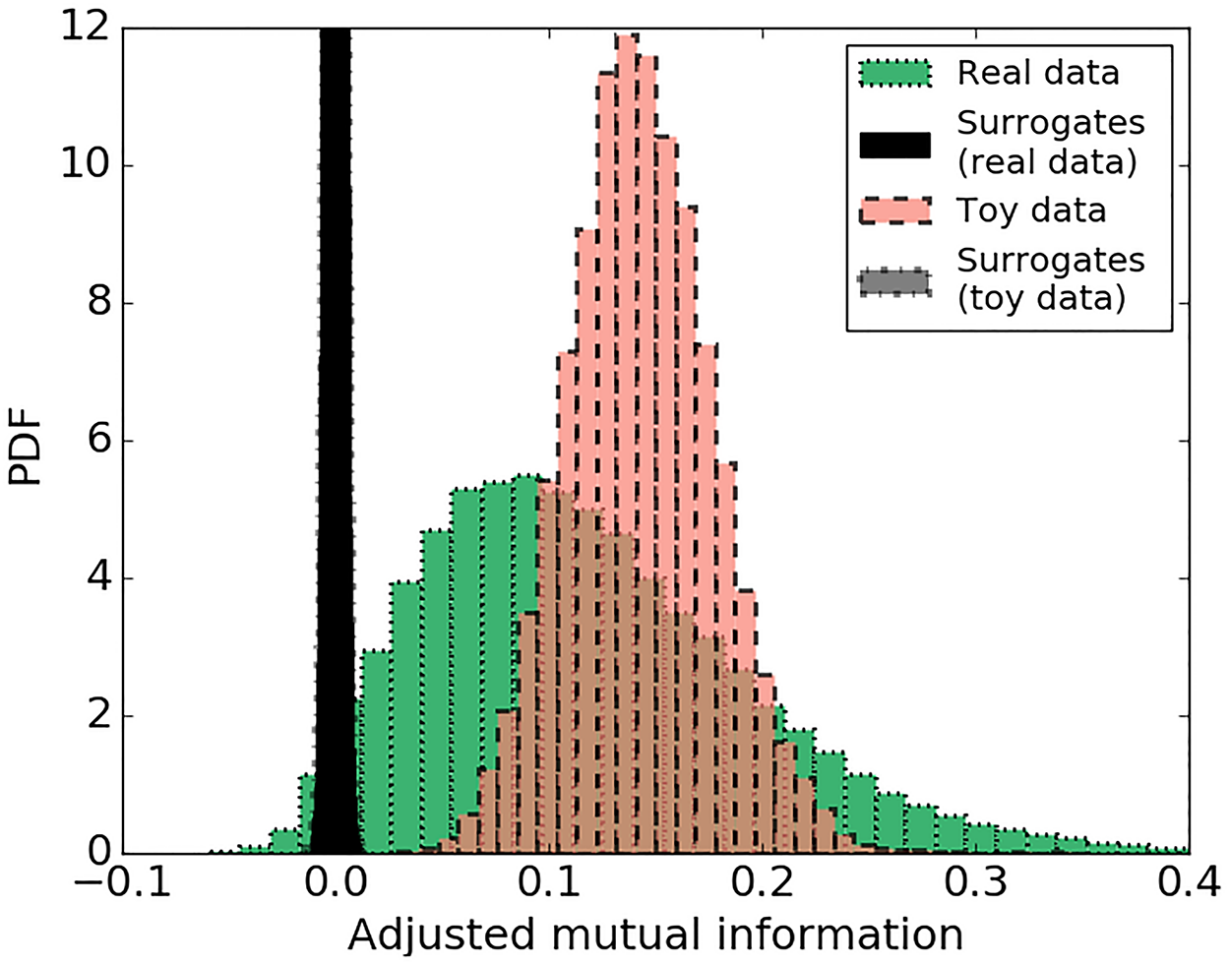
Probability density function of adjusted mutual information. AMI for real data (MAP and sub-band energy) (green) and its permuted surrogates (black); toy data (artificially created correlated signals) (red) and its permuted surrogates (grey). Distributions of surrogates are clipped.

Conventional MI has already been extensively tested and previously applied to biomedical signals, EEG in particular [30], [31], while AMI is a relatively new measure [29]. In order to check the statistical significance of the measures of interaction based on the MI concept, surrogate tests are conducted in practice [32], [33]. The surrogates are obtained by random permutation (shuffling) of the data, which preserves the frequency of the labels but destroys the coupling between the two sequences. The resultant measure of MI is indicative of interaction by chance only. By repeating the random shuffling (100 times in this work), the distribution of the chance-derived MI values is constructed and the statistical test is performed to assess whether the real MI value belongs to the distribution of the MI values obtained by chance. As the AMI measure explicitly accounts for chance, i.e. increasing the number of labels (bins) will not increase the value of AMI, therefore, there is no need to check the statistical significance of the obtained results with respect to chance. In order to illustrate this characteristic of AMI, artificial data were generated, which allows for the control of the different parameters, such as the level of noise and correlation between data while measuring the level of coupling. Two artificial sequences were simulated as a sum of sinusoids with and without random noise added (Fig 8). The surrogate test was performed on these sequences and presented in Fig 9.

It can be seen from the histograms in Fig 9 that the level of coupling measured by AMI for sequences with random interaction (surrogates) is centred on zero whereas the AMI values the for correlated data (red) is centred on 0.15. This confirms that AMI accounts for chance and that non-zero AMI values measure the inherent level of interaction in the two sequences. Fig 9 also shows the same plot for real data from the database (green). For every 30-minute window, AMI is calculated for the original sequence of MAP and EEG feature values and for permuted sequences. The distributions of the AMI values for both the real data and the toy data are clearly separated from corresponding surrogates, which implies the reliability of the calculated measure and indicates the presence of non-random interactions in the real data.

**Fig 10:**
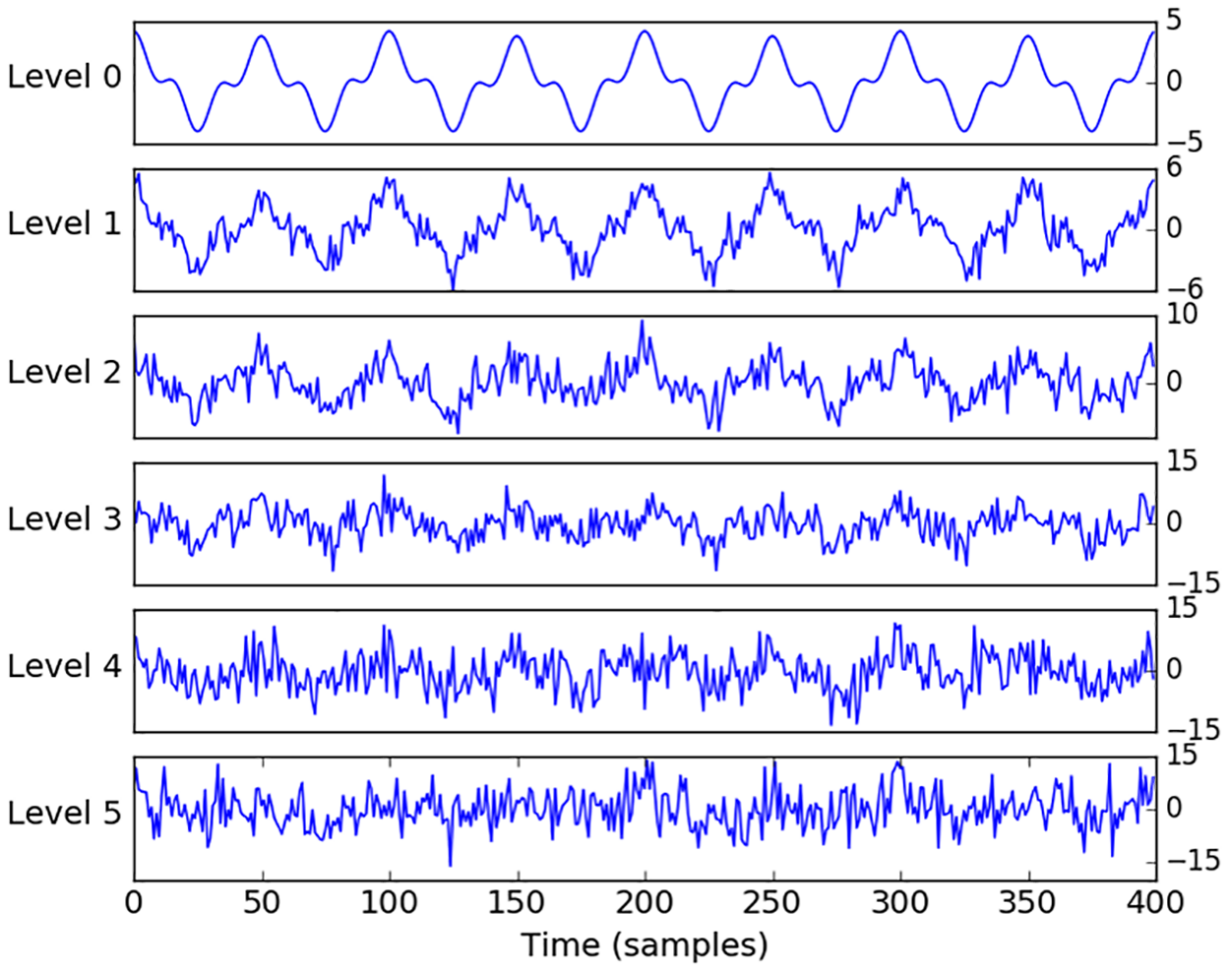
Artificially generated data with different levels of noise added.

**Fig 11:**
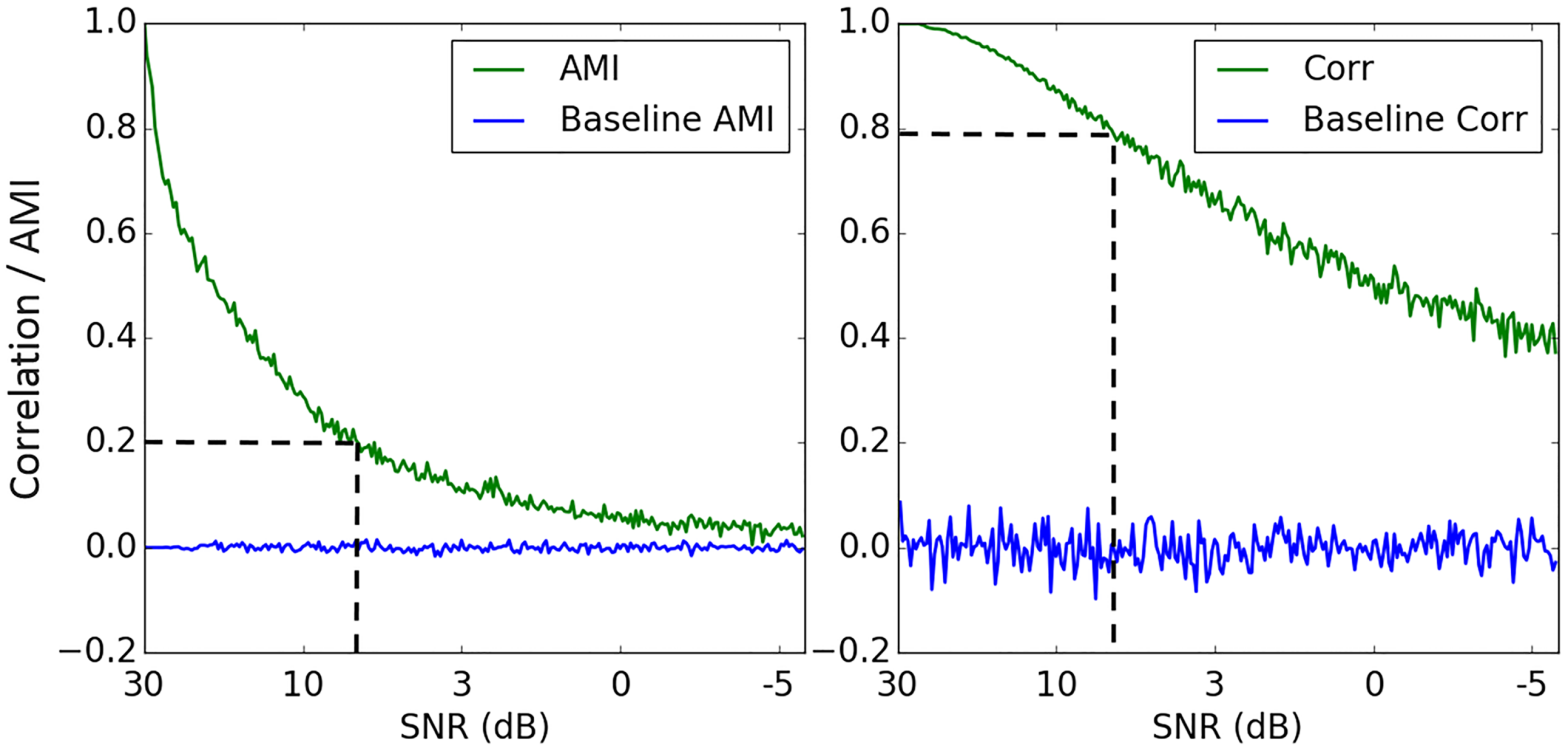
Effect of noise on AMI and correlation (green, bold). Baselines of zero coupling (blue) for both measures are represented as Pearson correlation and AMI between two Gaussian independent and identically distributed processes.

It can be seen from Fig 9 that AMI values are quite conservative, even for strongly correlated data. In order to check the sensitivity of the AMI measure to random noise and establish an intuitive connection with correlation, different noise levels were added to the artificially generated toy data as follows: *y* = *y*_0_ + *N*(0, *n*), where *n* is the standard deviation of random samples drawn from a Gaussian distribution (see Fig 10). A zero coupling baseline was set by measuring the interaction between two Gaussian independent and identically distributed (IID) processes. It can be seen from Fig 11, that the coupling of the two IID processes is centered around zero. This result shows an absence of interaction between two random sequences as measured by both AMI and correlation. We can also observe (Fig 11) that high levels of noise have a greater impact on AMI than correlation, where an AMI of 0.2 corresponds to a correlation coefficient of 0.8. Comparing Figs 11 and 9, it also can be seen that the operating range of AMI values for real data from 0.05 to 0.25 (Fig 9) corresponds to Pearson correlation coefficients of about 0.5–0.8 (Fig 11, dashed line). This justifies rather low and conservative values of AMI obtained even for clearly correlated data.

### Directionality of interaction: Transfer entropy

MI does not contain any directional information as it is a symmetric measure, where *MI*(***X***, ***Y***) = *MI*(***Y***, ***X***), and therefore it is not effective at predicting future events from the data or deriving the causality between two sequences. Transfer entropy (TE) is an extension of MI which takes into account the direction of informational flow, under the assumption that the underlying processes can be described by a Markov model [34]. TE allows the quantification of the exchange of information between two sequences, for each direction, by means of an introduced time lag in either one of the sequences. TE from a sequence ***X*** to another sequence ***Y*** is the amount of uncertainty reduced in future values of ***Y*** by knowing the past values of ***X***, given past values of ***Y***. The amount of information transferred from sequence ***X*** to sequence ***Y*** is denoted as *TE*_(***X***→***Y***)_ and is computed as follows:

$${TE}_{\left(X\to Y\right)}=\sum_{y_{t+u},y_{t}^{d_{y}},x_{t}^{d_{x}}}p(y_{t+u},y_{t}^{d_{y}}{,x}_{t}^{d_{x}})\text{log}\left(\frac{p(y_{t+u}|y_{t}^{d_{y}},x_{t}^{d_{x}})}{p(y_{t+u}|y_{t}^{d_{y}})}\right).$$ (9)

Here $p(y_{t+u}|y_{t}^{d_{y}},x_{t}^{d_{x}})=p(y_{t+u},y_{t},x_{t})/p(y_{t},x_{t})$ and $\;p(y_{t+u}|y_{t}^{d_{y}})=p(y_{t+u},y_{t})/p(y_{t})$, *t* is the point in time and *u* indicates the prediction time, e.g. *y*_t+u_ is the value of *Y* at time *t* + *u*. Values $y_{t}^{d_{y}}$ and $x_{t}^{d_{x}}$ are *d*_*y*_ - and *d*_x_- dimensional delay vectors. If the two processes are mutually independent there will be no transfer of information, therefore *p*(*y*_*t*+*u*_| *y*_*t*_) = *p*(*y*_*t*+*u*_| *y*_*t*_, *x*_*t*_) and *TE*_(***Y***→***X***)_ = *TE*_(***X***→***Y***)_ = 0.

Binning of data for computation of TE is no longer sensible as it ignores the neighbourhood relations in the continuous data and destroys the information about the absolute values of the original data [35] which is crucial for TE calculation. Examples where TE estimation fails due to the use of binned time series were previously reported in [36]. In [37] the estimation of TE was only properly obtained when using continuous data as opposed to its binned version. This problem has been solved by using the Kraskov-Stogbauer-Grassberger (KSG) nearest-neighbor based TE estimator for continuous data [38]. KSG is an improved box kernel estimator, which uses dynamically altered kernel width *r* which depends on the number of nearest neighbors. TE can be written using the representation of four Shannon entropies as:

$${TE}_{\left(X\to Y\right)}=S(y_{t}^{d_{y}},x_{t}^{d_{x}})-S(y_{t+u},y_{t}^{d_{y}},x_{t}^{d_{x}})+S(y_{t+u},y_{t}^{d_{y}})-S(y_{t}^{d_{y}}),$$ (10)

Every Shannon entropy (*S*(.)) is then estimated by the nearest-neighbor technique and the KSG estimator. The nearest-neighbor technique uses the statistics of the distance between neighboring data points in the embedding space. The KSG estimator uses a fixed number of neighbors for the search in the highest dimensional space and then projects the resulting distances to the lower dimensional space as the range to look for neighbors [38]. After adapting this technique to the formula with Shannon entropies, TE can be rewritten as:

$${TE}_{\left(X\to Y\right)}=\psi (K)+{\langle \psi (n_{{y_{t}}^{d_{y}}}+1)-\;\psi (n_{y_{t+u}{y_{t}}^{d_{y}}}+1)-\psi (n_{y_{t}}^{d_{y}}n_{x_{t}}^{d_{x}})\rangle }_{t},$$ (11)

where *ψ* is a digamma function and 〈.〉_*t*_ indicates an averaging over different time points. In this work the number of nearest neighbours was chosen to be *K* = 4, as recommended in [38] to balance bias, which decreases for larger *K*, and variance, which tends to increase for larger *K*. In order to find an embedding dimensions for MAP and EEG features we have used the measure of active information storage (AIS), which defines the past information of the process that can be used to predict its future [39]. AIS *A*_*X*_ for the sequence ***X*** is defined as the expected MI between the past state of the process $X_{t}^{d}$ (as *d* → ∞) and its next state *X*_*t*+1_:

$$A_{X}(d)=MI(X_{t}^{d},X_{t+1}).$$ (12)

Here *d* is an embedding dimension, which captures the underlying state of the process *X* for a Markov process of order *d*. TE was estimated using an open source toolbox JIDT [40]. When conducting statistical analysis for TE results, it is necessary to take into consideration the chosen values of the embedding dimensions e.g. the length of history we are checking in *Y* when trying to predict *X*. The reliability of *TE*_(*Y*→*X*)_, is tested against ${TE}_{\left(Y^{s}\to X\right)},$ where *Y*^*s*^ is a permuted surrogate, created by shuffling vectors $y_{t}^{d_{y}}$ [40], [41],[42]. As a result, the obtained surrogates preserve $p(x_{t+1}|x_{t}^{d_{x}})$, but not $p(x_{t+1}|x_{t}^{d_{x}},y_{t}^{d_{y}})$.

## Results

The results of the association between CRIB scores and the computed measures of coupling are presented in Table 2. Insignificant values of correlation were defined using the 95% CI obtained using the bootstrap method (random sampling with replacement).

**Table 2:** Correlation coefficient and 95% CI (in brackets) between CRIB score and coupling measures (correlation, coherence, AMI and TE) between MAP and four EEG sub-band powers.

	(EEG 0.3–3 Hz & MAP) vs CRIB	(EEG 3–8 Hz & MAP) vs CRIB	(EEG 8–15 Hz & MAP) vs CRIB	(EEG 15–30 Hz & MAP) vs CRIB
**Correlation**	NS	NS	NS	NS
**Coherence**	NS	NS	NS	NS
**AMI**	r: -0.57 (-0.78, -0.22)	NS	r: -0.42 (-0.68, -0.03)	NS
**TE (EEG to MAP)**	r: 0.428 (0.11, 0.68)	r: 0.44 (0.21, 0.66)	r: 0.416 (0.13, 0.67)	NS
**TE (MAP to EEG)**	NS	NS	NS	r: -0.436 (-0.68, -0.17)

Correlation coefficients were determined to be significant when their 95% CI excludes zero.

### Correlation and coherence

No statistically significant association was found between the CRIB scores and the level of linear coupling between MAP and all four EEG sub-band energies measured using both correlation and coherence.

### Adjusted mutual information

**Fig 12:**
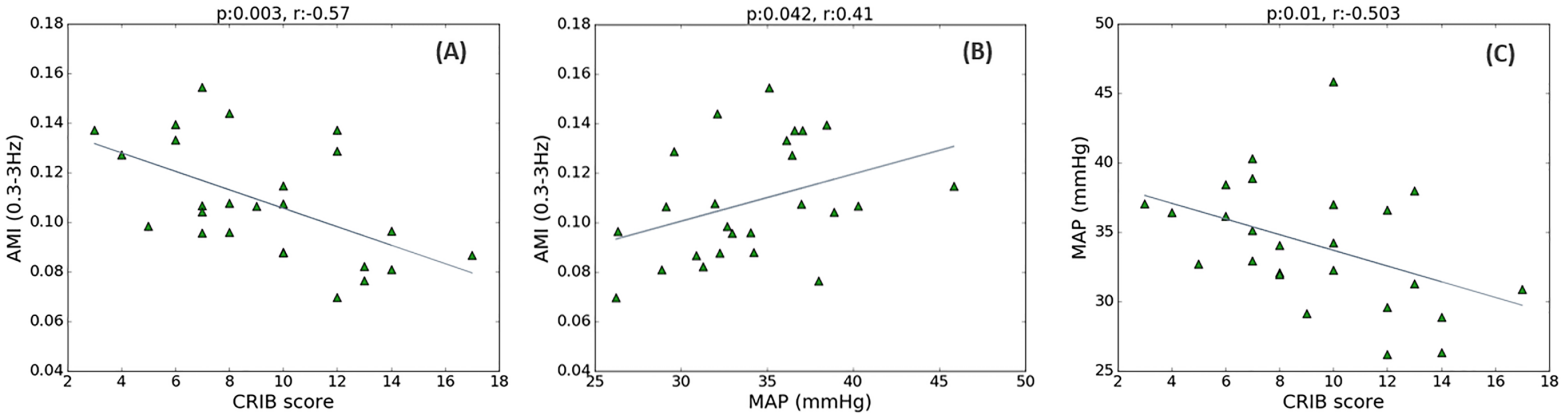
The relationship between CRIB, MAP and EEG energy. (A) CRIB score and AMI between MAP and EEG energy (0.3-3Hz); (B) MAP and AMI; (C) CRIB score and MAP. Every point on the scatter plots represents 1 newborn.

Fig 12 shows the association between the AMI, using sub-band energy 0.3–3 Hz, and the CRIB scores. As expected, a low MAP correlates with high CRIB scores, Fig 12C, where higher risks of mortality are associated with lower MAP values (*r = -0*.*503*, *p = 0*.*01*). This association could be indirectly associated to gestational age, as CRIB scores and MAP are both dependent on the GA. At the same time, the higher CRIB scores were not correlated with changes in any of the EEG energy bands (0.3–3 Hz: *r = 0*.*24*, *p = 0*.*2*; 3–8 Hz: *r = 0*.*08*, *p = 0*.*7*; 8–15 Hz: *r = -0*.*12*, *p = 0*.*6* and 15–30 Hz: *r = -0*.*2*, *p = 0*.*3*). It can also be seen from Fig 12A that the CRIB score has marginally higher correlation with the developed measure of interaction between signal dynamics, AMI, than with MAP (*r = -0*.*57*, *p = 0*.*003* vs *r = -0*.*503*, *p = 0*.*01*). A statistical test of the equality of the two correlation coefficients obtained from the same sample, with the two correlations sharing one variable (CRIB) did not show significant difference between them. The level of correlation between the CRIB score and the AMI for MAP with other EEG sub-band energies was significant only for 8–15 Hz sub-band (3–8 Hz: *r* = -0.3, *p* = 0.13; 8–15 Hz: *r* = -0.42, *p* = 0.04 and 15–30 Hz: *r* = -0.36, *p* = 0.08). There is also a statistically significant correlation between AMI and MAP for two sub-bands only (0.3–3 Hz: *r* = 0.41, *p* = 0.04; 3–8 Hz: *r* = 0.35, *p* = 0.09; 8–15 Hz: *r* = 0.38, *p* = 0.06; 15–30 Hz: *r* = 0.54, *p* = 0.004). Increase in MAP was associated with increasing GA *(p* = 0.001, *r* = 0.6*)* and an increase in EEG (15–30 Hz) spectral power *(r* = 0.541, *p* = 0.005*)*.

When considering data recorded during the first 24 hours of life of the preterm (Fig 2), the association between AMI for the sub-band energy 0.3–3 Hz and the CRIB score has improved with respect to the values computed from the whole recordings (*r* = -0.57, *p* = 0.003 vs *r* = -0.651, *p* = 0.001).

### Directionality of interaction: Transfer entropy

**Fig 13:**
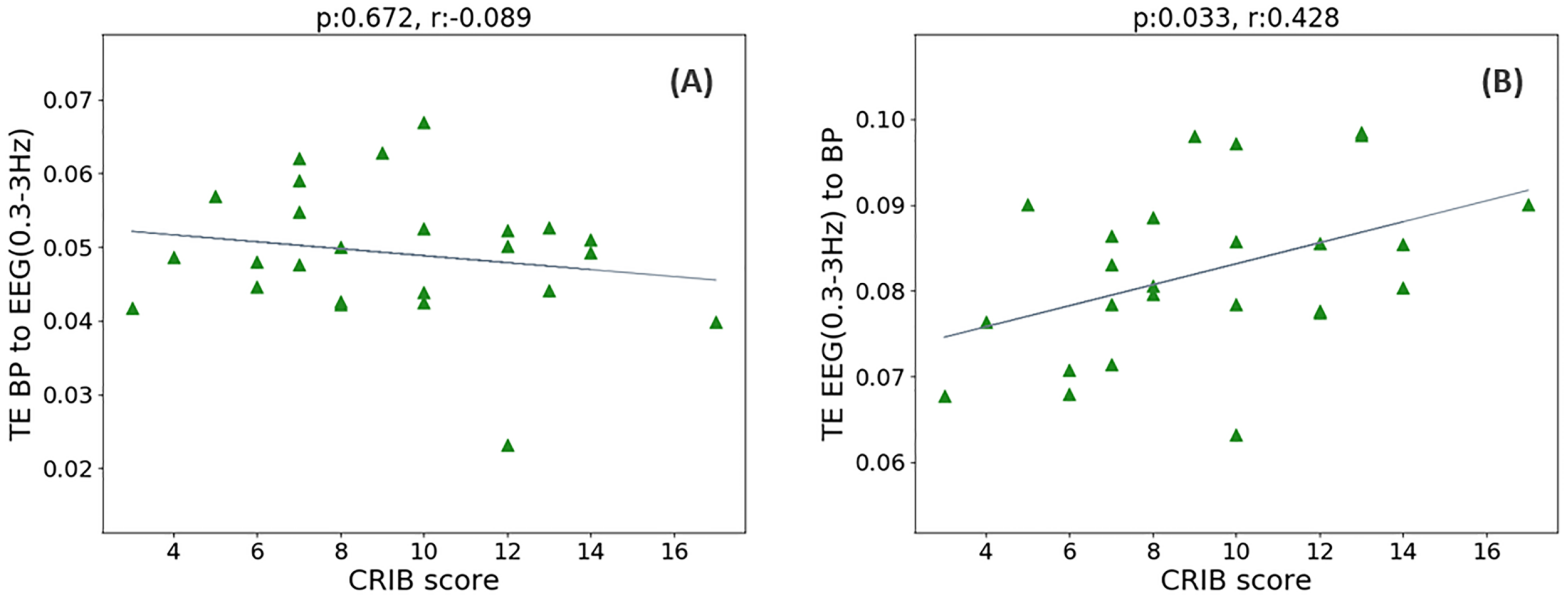
The relationship between the CRIB score and interaction between MAP and EEG energy. (A) CRIB score vs TE from MAP to EEG (0.3–3 Hz); (B) CRIB score vs TE from EEG (0.3–3 Hz) to MAP.

**Fig 14:**
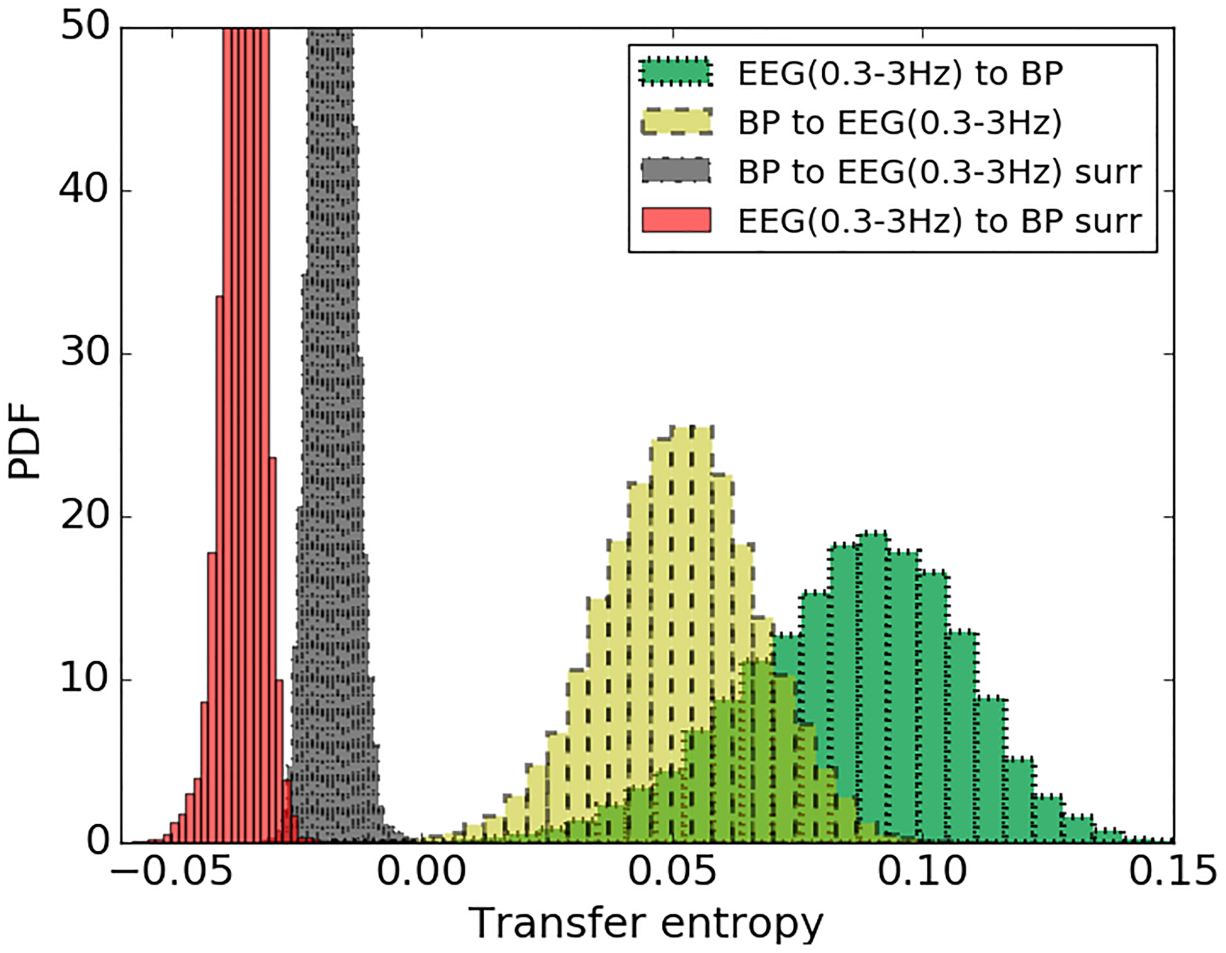
The distribution of TE values for real data and randomly permuted surrogates. TE is quantified from MAP to EEG (0.3–3 Hz) for real data (yellow) and corresponding randomly permuted surrogates (black). TE from EEG (0.3-3Hz) to MAP for real data (green) and its randomly permuted surrogates (red).

While AMI is focused on the detection of the significant coupling between sequences, transfer entropy (TE) also detects the direction of coupling. As represented in Fig 13, no association was found between TE (MAP to EEG (0.3–3 Hz)) and CRIB score (*r* = -0.089, *p* = 0.672), however the transfer of information in the opposite direction, from EEG (0.3–3 Hz) to MAP, showed an association with CRIB scores (*r* = 0.428, *p* = 0.033). Results of TE for the other three EEG sub-band powers are represented in Table 2. From Fig 14 it can be seen that TE of real data is separated from its corresponding surrogates. This indicates the reliability of the obtained TE values. At the same time TE from MAP to EEG are lower than corresponding values of TE from EEG to MAP.

## Discussion

### Nonlineal relationship between MAP and EEG sub-band powers

The main finding of the present study is related to nonlinear measures of interaction between cerebral activity and MAP for preterm neonates. A statistically significant association of the CRIB scores with AMI is observed for low frequency (0.3–3 Hz) sub-band energy of EEG. Maturational features for neonatal EEG vary across gestational ages. Most of the preterm EEG power is known to be concentrated in the lower frequencies. Delta (0–3.5 Hz) activity is a major characteristic of the preterm EEG that evolves as the infant matures and disappears between 38 and 42 weeks of gestation [25].

The obtained correlation of CRIB with AMI is higher than that of CRIB with MAP (*r* = -0.57, *p* = 0.003 vs *r* = -0.503, *p* = 0.01), although the difference was not statistically significant. However, it is worth emphasising that AMI is independent of the absolute values of both MAP and EEG energy and measures only the coupling between signal dynamics. Thus, the measure of dynamic interaction correlates with the median MAP per baby. We found lower levels of coupling at lower values of MAP. This indicates that low BP affects the interaction measured by AMI as do poor CRIB scores. Additionally, the results of simulation and surrogate tests which were used to detect random coupling, support the reliability of the obtained AMI values.

From Fig 2 it can be seen that every preterm neonate has different duration and timing of the recordings. When considering EEG and BP data during first 24 hours after birth, an association between AMI (0.3–3 Hz) and CRIB score has improved (*r* = -0.57, *p* = 0.003 vs *r* = -0.651, *p* = 0.001). This results indicate that the coupling between cerebral activity and BP is more sensitive to the risk index of the preterm during the first hours of life.

### Linear relationship between MAP and EEG sub-band power

In order to measure the linear association between MAP and brain activity, coherence and Pearson correlation were applied. Unlike the nonparametric Spearman’s rank correlation coefficient, which measures monotonic association between two variables, Pearson correlation measures linear coupling. To remove insignificant correlations known to occur because of chance, we used a surrogate data procedure by contrasting with shuffled surrogates. Linear measures of interaction such as correlation and coherence have been previously applied to adult physiological signals [43] including NIRS, EEG, ECG and BP where the analysis has been conducted on preselected 5-minute epochs from 19 subjects. In our work after discarding insignificant correlation coefficients (Fig 4) using surrogate tests, both correlation and coherence measures have indicated that these linear measures of interaction between brain activity and MAP failed to find an association with the underlying illness severity scores of the preterm infant.

Brain function is known to be a complex system of nonlinear processes and therefore it is likely that nonlinear methods would be more appropriate when measuring the interaction between EEG and MAP. The results of this study showed that a nonlinear method of coupling between MAP and EEG features measured using AMI is more sensitive to noise compared with the linear correlation method (Fig 11). According to [44] nonlinear measures are indeed very sensitive to noise and linear methods sometimes present better properties in this sense [45]. However, both linear and nonlinear approaches assess different aspects of the interdependence between the signals and provide a more comprehensive picture of the analysed data. Therefore, it is a good practice to use both methods to ensure that all the information available from the signals has been obtained and properly analysed with statistical and reliability tests (surrogates).

### Directionality of interaction

The detection of the presence of a dominant direction for the coupling between physiological systems can also provide an insight into their mutual interdependency. TE was previously used for establishing directed information structure between brain regions [46]. In the area of cardiovascular physiology this technique was utilized to define causal relationships that explain sources of variability in the regulation of cerebral hemodynamics [47]. In this study, the TE measure is used to provide an indication of the causal relationship of processes that occur between brain activity and MAP with respect to the illness risk of the preterm infant. The TE results passed the surrogate test which indicates that the directionality in the MAP and EEG sequences is present beyond the level of chance. The higher values of TE from EEG to MAP in comparison with the opposite direction indicate greater information transfer from EEG to MAP. The strength of this directionality apart from being greater, also correlates with the CRIB II scores for the first three EEG sub-band powers as shown in Table 2. This may indicate that sicker preterm infants have a higher level of information flow from brain activity to MAP for a wide range of frequencies (lower than 15 Hz).

The neuronal activation followed by hemodynamic changes has been previously reported in [48], [49]. At the same time it has been reported in [50], [51] that changes in cerebral oxygenation assessed by NIRS are likely to precede changes in EEG; in [50], the causality is represented by higher TE values from NIRS to EEG. Unlike the previous study where the EEG was recorded using only the C3-C4 channel, in this study eight bipolar EEG channels were incorporated, which enables a better coverage of the preterm brain [25]. Additionally, in our study the EEG was analysed through four sub-band energies from long duration unedited signals, whereas a single root mean square measure of EEG energy was used in [50]. Moreover, the interaction between EEG and MAP was explored in the context of CRIB scores, whereas in [50] the interaction was measured between EEG and NIRS under a sedation protocol. These differences make it difficult for a direct comparison of the results. The physiological mechanism of autoregulation for premature babies is not fully understood and to the best of our knowledge this is the first study to investigate the interaction and the directional information flow between MAP and EEG for preterm neonates. Therefore, in order to have a better understanding of physiological mechanism of connectivity between brain and MAP further studies are warranted.

### General discussion

**Fig 15:**
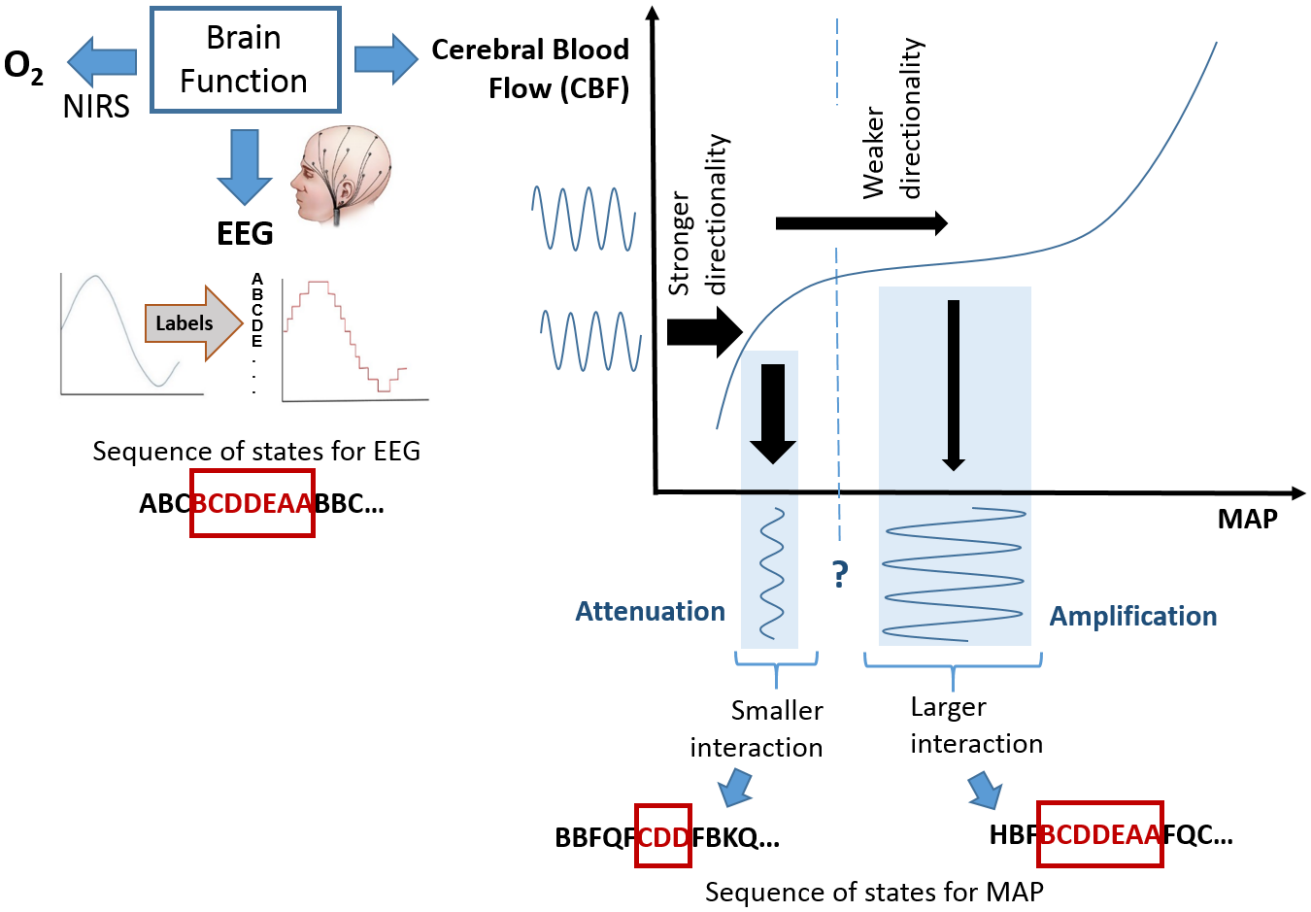
Schematic representation of the coupling between EEG and MAP using the autoregulation curve. The plateau of the autoregulation curve is used as a benchmark of normal brain function. The MAP and EEG are represented by the sequence of states denoted with letters. A higher level of interaction is implied by a longer overlap in the sequences (same patterns). When the MAP falls below an unknown threshold, the dynamic interaction between EEG and MAP changes. A higher risk of mortality which is represented with higher CRIB scores is shown to be associated with a smaller interaction between EEG and MAP and a stronger directionality of this interaction (from EEG to BP).

The results obtained in this study allow us to hypothesise that the stronger coupling between brain activity and MAP, as quantified by AMI, is related to the physiological status of a preterm (Fig 12A). At the same time, stronger directionality in the interaction is associated with an increased risk of mortality (Fig 13B). This hypothesis is schematically represented in Fig 15 based on the cerebral autoregulation curve. Here, the functioning cerebral autoregulation plateau which represents normal physiological wellbeing, corresponds to a stronger interaction between EEG and MAP (higher AMI values) and weaker EEG-to-MAP directionality (TE values). If it is argued that the overall status of the infant is affected by hypotensive periods, then the problem becomes one of comparing the MAP with some lower threshold, such as MAP = GA. However, there is not one common threshold for every infant. In this work, it is proposed for a particular infant, that when the MAP falls below this unknown threshold that the dynamic (rather than static) interaction changes. Therefore, identifying the change in slope of the autoregulation curve, through the proposed measures of dynamic interaction, can be used as a proxy for identifying the threshold.

The area of hypotension remains one of the most challenging in the newborn care. Its diagnoses and subsequent management for the very preterm infant remains controversial [6], [7]. Several studies have attempted to determine normal ranges of BP for the newborn. However, such definitions are not consistent and vary between studies [1], and increase with increasing GA [12], which complicates the diagnosis of hypotension and efficiency of treatment processes. It is known that hypotension may cause decreased cerebral perfusion and a number of studies have previously attempted to establish the relationship between BP and brain perfusion. Unfortunately there is still no direct ways to measure cerebral blood flow and as a result NIRS and EEG are usually utilized as surrogate measures of brain health, where EEG has been shown to be a good predictor of early neonatal outcome [52]. In order to better understand the interrelation between brain function and BP, some studies have also incorporated measures of cardiac output [12], [13]. In this study we hypothesise that during periods of low BP, EEG activity would change, which may lead to a change in coupling between BP and brain activity and therefore may be indicative of newborn wellbeing. The association between BP and EEG activity in neonates is complex and can be influenced by blood supply to the brain as well as autoregulatory mechanisms. It was previously reported that cerebral autoregulation is poorly developed in the preterm and is influenced by many factors [53]. This is supported by a study [54] where forty preterm infants were assessed but no statistically significant relationship between BP and cerebral electrical activity was identified. Similar to (12, 53) we observed an increase in MAP with increasing GA (*r* = 0.61, *p* = 0.001). At the same time an increase in EEG (15–30 Hz) spectral power was also associated *(r* = 0.54, *p* = 0.005*)* with increasing MAP. No changes in absolute spectral powers were associated with the GA. In contrast, other studies reported changes in absolute and relative spectral powers with increased postmenstrual age (maturation) (15, 41, 42). There may be a number of reasons for this. It is known that relative power, which is not considered in the current study, is better at capturing the maturational changes in the preterm brain than absolute power, as this measure is more sensitive to the changes in EEG discontinuity. Another reason could be related to different EEG processing routines and to procedures which were used to select EEG segments. For instance, in [55] spectral analysis was performed on only eight 4-second epochs from each recording. Study [56] has also analysed preselected short EEG epochs with a duration of only 10-seconds. In contrast, in our study, spectral analysis was performed on the continuous multi-channel unedited EEG recordings from 25 subjects with a total duration of 957 hours.

Quantitative analysis of EEG data can provide valuable information about cerebral activity. However, analysis of EEG contaminated by artefacts may lead to spurious results. In this study the process of EEG and BP artefact identification was addressed in several stages as described in the “Preprocessing and feature extraction: EEG and BP” section. Even if the some artefacts survive the previous “filters”, the median across temporal measures of interaction is computed across the entire subject recording (median per subject of 37 hours, IQR = 24 to 48 hours), ensuring the coupling measures are robust against extreme values which may possibly have been caused by artefacts.

Holm-Bonferroni correction for the multiple comparison (n = 5) showed that in order to be significant at alpha level of 0.05, the first-ranked (smallest) *p* value needs to be smaller than 0.01. After the correction is applied, the association of AMI between MAP and EEG 0.3–3 Hz and CRIB score remained statistically significant which is the main result of the study (Table 2). This study, however, is of exploratory nature and aims at open-ended hypothesis generation. Several researchers have recently argued that *p* values lose their meaning in exploratory analyses due to an unknown inflation of the alpha level [57], [58]. This allows *p* values to serve as a guide for the hypothesis to be tested in further confirmatory research.

The results of the paper indicate that the physiological reaction to the changes of BP is associated with lower risks of the preterm. This finding can potentially contribute towards the generation of a hypothesis in the field of hypotension management and the interrelation between cerebral activity and BP for preterm neonates. Two channels of EEG are now routinely used in infants who are suspected of having brain injury. Therefore, we anticipate that a module in a bedside monitor incorporating the algorithm to compute and visualise the measure of interaction between BP and cerebral activity would be feasible, providing a real time decision support for more efficient management of hypotension in preterm neonates.

### Limitations

Preterm cortical activity can be characterised by a number of maturation features [25], such as continuity, sleep states and others. In this study we assessed the first days of life of the preterm only, where every infant was represented by a single summary measure (median across the recording). As a result, this did not allow us to investigate the possible impact of cyclical activity, such as sleep states, on the coupling across time.

In order to better represent the population of preterm neonates, further confirmatory research should be conducted on a larger cohort of preterm neonates with a wider range of CRIB II scores. This, however, may be a challenging task, as a continuous multichannel long EEG recordings are very difficult to obtain for the population of preterm neonates. These babies are extremely vulnerable and any intervention should be agreed with neonatologist. At the same time neonates with high CRIB II score are very sick and it is difficult to get a permission for such an intervention.

## Conclusions

This is the first study that investigates the relationship between short-term dynamics in BP and EEG energy in the preterm on a large dataset of continuous multi-channel unedited EEG recordings. The coupling between EEG and BP is computed using both linear and nonlinear measures for 25 preterms. Our findings suggest that nonlinear measures of interaction are more suitable when measuring coupling between the complex system of brain function and BP. The results are tested with surrogate reliability tests and contrasted with the preterm wellbeing represented by the CRIB II score. The results reported in this study have indicated that a higher risk of mortality for the preterm is associated with a lower level of nonlinear interaction between EEG and MAP which is measured by AMI. The computation of the proposed measure of interaction is independent of absolute values of MAP and GA-based thresholds. It has been shown that higher CRIB scores are also associated with higher levels of information flow from EEG-to-MAP as measured by TE. This allows us to hypothesise that normal wellbeing of a preterm neonate can be characterised by a strong nonlinear coupling between brain activity and MAP, whereas the presence of weak coupling with distinctive directionality of information flow may be associated with an increased risk of illness severity in preterms.

## Supporting information

S1 Data Database details. (7Z)
